# *ANXA9* gene expression in colorectal cancer: A novel marker for prognosis

**DOI:** 10.3892/ol.2014.2477

**Published:** 2014-08-25

**Authors:** NORIKATSU MIYOSHI, HIROFUMI YAMAMOTO, KOSHI MIMORI, SHINYA YAMASHITA, SUSUMU MIYAZAKI, SUMIKO NAKAGAWA, HIDESHI ISHII, SHINGO NOURA, MASAYUKI OHUE, MASAHIKO YANO, YUICHIRO DOKI, MASAKI MORI

**Affiliations:** 1 Department of Surgery, Osaka Medical Center for Cancer and Cardiovascular Diseases, Osaka, Osaka 537-8511, Japan; 2 Department of Gastroenterological Surgery, Osaka University Graduate School of Medicine, Suita, Osaka 565-0871, Japan; 3 Department of Molecular and Cellular Biology, Division of Molecular and Surgical Oncology, Medical Institute of Bioregulation, Kyushu University, Beppu, Õhita 874-0838, Japan; 4 Department of Surgery, National Hospital Organization Kure Medical Center, Kure, Hiroshima 737-0023, Japan; 5 Department of Surgery, Osaka General Medical Center, Osaka, Osaka 558-8558, Japan; 6 Department of Frontier Science for Cancer and Chemotherapy, Osaka University, Suita, Osaka 565-0871, Japan

**Keywords:** *ANXA9*, prognosis, colorectal cancer

## Abstract

Annexin A9 (*ANXA9*) is involved with the interaction with membrane phospholipids in a Ca^2+^-dependent manner. A previous study has shown that *ANXA9* expression is associated with bone metastasis in breast cancer, whereas its significance in colorectal cancer (CRC) is unknown. The present study was comprised of 100 patients who underwent surgery for CRC. The correlation between gene expression and the clinical parameters of the patients was assessed. Patients with high *ANXA9* expression were statistically susceptible to a relatively worse prognosis, and those with low *ANXA9* expression showed improved overall survival compared with those with high expression. In conclusion, the present data suggests that *ANXA9* expression is a prognostic factor in CRC patients.

## Introduction

In developed countries where the aging population is increasing, cancer is one of the most prominent illnesses in terms of public welfare and health. One in four mortalities in the United States, for example, is due to cancer (1). In the United States, the incidence of CRC has increased significantly in recent years, based on changing lifestyles. CRC is one of the most prominent causes of mortality from neoplastic disease in Japan. Distant metastases, such as liver or lung metastases, are the major cause of mortality in CRC (2). The ability to identify the genes responsible for CRC development and progression and the ability to understand the associated clinical significance are vital for diagnosing and treating CRC sufficiently. The characterization of key molecules has great potential with regard to the generation of novel approaches for the treatment of CRC.

The annexins are a family of well-conserved proteins, characterized by the ability to interact with membrane phospholipids in a Ca^2+^-dependent manner (3). The structures of all annexins contain type II Ca^2+^ sites that are located in the protein core domains, built of four or eight homologous segments known as annexin repeats (4,5). In breast cancer, *ANXA9* has been reported as a gene that is associated with the relapse in bone (6). However, the association between *ANXA9* expression and CRC remains unknown.

The aim of the present study was to analyze the correlation between *ANXA9* expression levels in the CRC tissues of patients and the clinicopathological factors, and to investigate the possible functions of the gene in the tumorigenesis and metastasis of CRC.

## Materials and methods

### Clinical tissue samples

In total, 100 patients (61 males and 39 females) with CRC were registered and underwent curative surgery for resection of CRC and distant metastases, if present, at the Medical Institute of Bioregulation at Kyushu University (Beppu, Ohita, Japan) and the Department of Gastroenterological Surgery, Osaka University Graduate School of Medicine (Suita, Osaka, Japan) between 1994 and 2003. None of the patients received chemotherapy or radiotherapy prior to surgery. Primary CRC specimens and adjacent normal colorectal mucosa samples were obtained from the patients following the receipt of written informed consent. This study was approved by the ethics committee of Osaka University Graduate School of Medicine (Osaka, Japan). The surgical specimens were fixed in formalin, processed through graded ethanol and embedded in paraffin. The sections were stained with hematoxylin and eosin, and Elastica van Gieson (Merck Millipore, Billerica, MA, USA) stains, and the degree of histological differentiation, lymphatic invasion and venous invasion was examined. Additionally, sections from all specimens were frozen in liquid nitrogen immediately after resection and kept at −80°C until RNA extraction. Subsequent to surgery, the patients underwent follow-up blood examinations to assess the tumor markers, serum carcinoembryonic antigen (CEA) and cancer antigen (CA19-9), and imaging modalities, such as abdominal ultrasonography, computed tomography and chest X-rays, were performed every 3 to 6 months. Post-operatively, the stage III and IV patients received 5-fluorouracil-based chemotherapy for six months [mFOLFOX6; (oxaliplatin, 85 mg/m^2^, 5-fluorouracil 2800 mg/m^2^, for 2 weeks, for 12 courses), UFT (300 mg/m^2^/day × 28 days/5 weeks, for 5 courses), capecitabine (2500 mg/m^2^/day × 14 days/3 weeks, for 8 courses), or TS-1 (80 mg/m^2^/day × 28 days/6 weeks, for 4 courses)], whereas the stage I and II patients principally received no chemotherapy. All therapies were performed according to the Japanese Society for Cancer of the Colon and Rectum guidelines (7). Clinicopathological factors were assessed according to the tumor node metastasis (TNM) classification of the International Union Against Cancer (8).

### RNA preparation and expression analysis

Total RNA was prepared using TRIzol reagent (Invitrogen, Carlsbad, CA, USA) or with DNase using a modified acid guanidium-thiocyanate-phenol-choroform procedure (9). Reverse transcription was performed with SuperScriptII (Life Technologies, Carlsbad, CA, USA) or by the methods reported previously (10). A 242-bp *ANXA9* fragment was amplified. Two human *ANXA9* oligonucleotide primers for the polymerase chain reaction (PCR) were designed as follows: Forward, 5′-TGAGCCCAATTACCAAGTCC-3′ and reverse, 5′-GTTCAGCCAAACACGGAAAT-3′. The forward primer is located in exon 13 and the reverse primer in exon 14. A PCR kit (TaKaRa Ex Taq; Takara, Kyoto, Japan) on GeneAMP PCR System 9600 (PE Applied Biosystems, Foster City, CA, USA) was used to perform 35 cycles of PCR with the following parameters: 95°C for 40 sec, 45°C for 40 sec and 72°C for 60 sec. An 8-μl aliquot of each reaction mixture was size-fractionated in a 1.5% agarose gel and visualized with ethidium bromide staining. To ensure that the RNA was not degraded, a PCR assay with primers specific for the glyceraldehyde-3-phosphate dehydrogenase (*GAPDH*) gene was performed for 1 min at 95°C, 1 min at 56°C and 1 min at 72°C for 30 cycles. The *GAPDH* primers were as follows: Forward, 5′-TTGGTATCGTGGAAGGACTCA-3′ and reverse, 5′-TGTCATCATATTGGCAGGTT-3′, and produced a 270-bp amplicon. Complementary DNA from the Human Reference Total RNA (Clontech, Palo Alto, CA, USA) was studied concurrently as a source of positive controls. For quantitative assessment, reverse transcription-quantitative PCR (RT-qPCR) was performed using a LightCycler FastStart DNA Master SYBR Green I kit (Roche Diagnostics, Tokyo, Japan) for cDNA amplification of *ANXA9* and *GAPDH*. The amplification protocol consisted of 35 cycles of denaturation at 95°C for 10 sec, annealing at 60°C for 10 sec and elongation at 72°C for 10 sec. The products were then subjected to a temperature gradient from 55°C to 95°C with continuous fluorescence monitoring to produce a melting curve of the products. The expression ratios of the *ANXA9* mRNA copies in the tumor and normal tissues were calculated following normalization against *GAPDH* mRNA expression.

### Statistical analysis

The *ANXA9* expression levels between the CRC and normal colorectal mucosa (normal tissue) samples, and the association between *ANXA9* expression and the clinicopathological factors were analyzed with the χ^2^ test. Kaplan-Meier survival curves were plotted and compared with the generalized log-rank test. Univariate and multivariate analyses to identify prognostic factors were performed using Cox’s proportional hazard regression model. The values in the *in vitro* assays were analysed with Wilcoxon’s rank test. All tests were analyzed with JMP software (SAS Institute, Cary, NC, USA). P<0.05 was considered to indicate a statistically significant difference.

## Results

### Expression of ANXA9 in clinical tissue specimens

**Figure 1:**
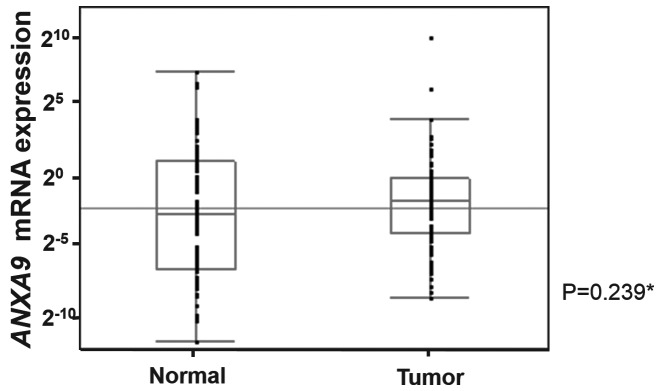
*ANXA9* mRNA expression in clinical tissue specimens. Reverse transcription-quantitative polymerase chain reaction on 100 paired clinical samples showed that 56 of these cases (56.0%) exhibited higher levels of *ANXA9* mRNA in the tumor samples compared with the paired normal tissues. The mean *ANXA9* mRNA expression level in the tumor tissues (normalized by *GAPDH* gene expression) was not significantly different compared with that of the corresponding normal tissues (P=0.239, Wilcoxon’s rank test). *ANXA9*, annexin A9; *GAPDH*, glyceraldehyde-3-phosphate dehydrogenase.

RT-qPCR analysis was performed with primary CRC tissues and samples from adjacent normal colorectal regions. *ANXA9* expression was calculated as *ANXA9*/*GAPDH* expression for each tumor or normal tissue sample (Fig. 1). The mean expression level in the tumor tissues was found to be larger than that of the normal tissues, and there was a significant difference between the tumor and normal tissues subsequent to dividing the samples into two groups according to the mean expression value of the tumor and normal tissues (P=0.047) (Table I). In the following analyses, *ANXA9* expression normalized by *GAPDH* expression in the tumor tissue was calculated following division by *ANXA9* expression level in the normal tissue.

### Expression of ANXA9 and clinicopathological characteristics

For the clinicopathological evaluation, experimental samples were divided into two groups according to the expression status. Patients with values >1 (*ANXA9* expression level in the tumor tissue was larger than that of the corresponding normal tissue) were assigned to the high expression group and the others were assigned to the low expression group. Clinicopathological factors associated with the *ANXA9* expression status of the 100 patients are summarized in Table II. The data indicated that *ANXA9* expression was not significantly correlated with these clinicopathological factors.

### Association between ANXA9 expression and prognosis

**Figure 2:**
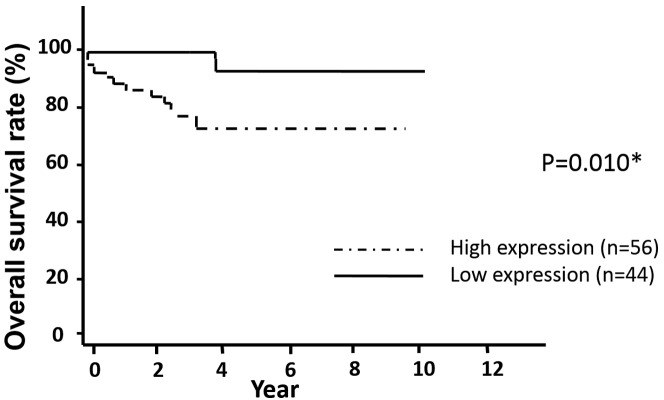
Overall survival curves based on *ANXA9* mRNA expression status of colorectal cancer patients. The post-operative overall survival rate was significantly lower in the patients in the high expression group compared with the low expression group (P=0.010, log-rank test). *ANXA9*, annexin A9.

The data shows that the post-operative overall survival rate was significantly lower in the patients in the high expression group compared with that in the low expression group (P=0.010) (Fig. 2). The median follow-up time was 3.83 years. Table III shows the results of the univariate and multivariate analyses for factors associated with overall survival. The univariate analysis showed that age (P=0.010), tumor invasion (P=0.002), lymph node metastasis (P=0.001), lymphatic invasion (P=0.012), venous invasion (P=0.026), metastasis (P<0.001) and *ANXA9* expression (P=0.004) were significantly correlated with overall survival. The multivariate regression analysis indicated that the *ANXA9* high expression group (hazard ratio, 3.13; 95% confidence interval, 1.32–12.86; P=0.007), tumor invasion (hazard ratio, 2.21; 95% confidence interval, 1.09–5.02; P=0.026) and distant metastasis (hazard ratio, 10.82; 95% confidence interval, 3.08–35.36; P=0.001) were independent predictors of overall survival.

## Discussion

The annexin gene family was discovered in 1984 and the members were isolated in the presence of calcium, which served as a substrate for the epidermal growth factor receptor/kinase (11,12). ANXA9, initially termed annexin 31, is a protein that is believed to function in the organization and regulation of membrane/cytoskeleton linkage (13). The gastrointestinal cancer cell line, HepG2, expresses ANXA9 protein, which can be detected by A9-specific antibodies (3). The expression of annexin has been reported in *Xenopus*, *Drosophilia*, *Dictyostelium*, *Caenorhabditis elegans*, *Neurospora*, *Giardia* and all plants (14–16). However, the role of annexin in cancer has not been clearly defined and the biological analysis remains incomplete.

The present study showed that *ANXA9* expression is an independent prognostic factor for CRC. This suggests that tumor malignancy correlates with *ANXA9* expression and that this may also affect the values of other prognostic factors in multivariate analysis, such as distant metastasis, which was significant in the univariate analysis. *ANXA9* expression was a significant prognostic factor reflecting overall survival and distant metastasis. To the best of our knowledge, the present study is the first to show *ANXA9* as a statistically significant predictor for CRC prognosis following curative resection, as well as other reported factors (17). The present results suggest that an *ANXA9*-dependent pathway may be involved in the progression of CRC.

The prediction of recurrence and metastases following curative surgical resection aid the determination of the necessity for intensive follow-up and adjuvant CRC therapy (18–20). While certain patients respond well to CRC treatment, others do not, therefore individualized predictions and strategies with higher precision are required for treating metastasis (21). In the present study, the clinicopathological analysis revealed that CRC patients with low levels of *ANXA9* expression showed an improved prognosis for overall survival compared with those patients with high levels of expression. The data indicates that *ANXA9* is a presumptive novel predictor of CRC prognosis.

Several adjuvant chemotherapies are valuable in specific stages of CRC and indicate the usefulness of less invasive surgery for the disease (17–20,22–28). For these cases, an informative prognostic marker, which is independent from the traditional TNM classification and contributes to diagnoses and treatments, is extremely important. The present data indicate the candidacy of *ANXA9*. While improved pre-operative and post-operative treatments, such as chemotherapy and radiotherapy combined with surgery, have contributed to the reduction of recurrences of CRC, half of all cases eventually metastasize despite systemic chemotherapy followed by surgery (29). Adjuvant chemotherapy for CRC is preferred in highly suspicious cases of recurrence. In these cases, *ANXA9* analysis may aid in the predictions and treatment of patients with a poor prognosis.

## Figures and Tables

**Table I:** *ANXA9* mRNA expression in primary CRC specimens and normal colorectal mucosa samples.

*ANXA9* expression	Primary CRC	Normal mucosa	P-value
<Mean	41	55	0.047*
≥Mean	59	45	

* Statistically significant, P<0.05.

*ANXA9*, annexin A9; CRC, colorectal cancer.

**Table II:** Clinicopathological factors of 100 colorectal cancer patients with high (n=56) and low (n=44) levels of *ANXA9* mRNA expression.

Factors	Low expression, n (%)	High expression, n (%)	P-value
Age, years			
<68	25 (56.8)	27 (48.2)	0.392
≥68	19 (43.2)	29 (51.8)	
Gender			
Male	24 (54.5)	37 (66.1)	0.244
Female	20 (45.5)	19 (33.9)	
Histological grade			
Well-moderate	41 (93.2)	55 (98.2)	0.202
Poor	3 (6.8)	1 (1.8)	
Tumor size, mm			
<30	8 (18.2)	9 (16.1)	0.780
≥30	36 (81.8)	47 (83.9)	
Tumor invasion			
Tis	4 (9.1)	3 (5.4)	0.316
T1	7 (15.9)	6 (10.7)	
T2	9 (20.4)	6 (10.7)	
T3	19 (43.2)	28 (50.0)	
T4	5 (11.4)	13 (23.2)	
Lymph node metastasis			
N0	31 (70.5)	33 (58.9)	0.233
N1–2	13 (29.5)	23 (41.1)	
Lymphatic invasion			
Absent	20 (45.5)	26 (46.4)	0.824
Present	24 (54.5)	30 (53.6)	
Venous invasion			
Absent	33 (75.0)	42 (75.0)	1.000
Present	11 (25.0)	14 (25.0)	
Metastasis			
M0	38 (86.4)	50 (89.3)	0.655
M1	6 (13.6)	6 (10.7)	
UICC stage			
0-I	17 (38.6)	13 (23.2)	0.221
II	13 (29.5)	18 (32.1)	
IIIA	4 (9.1)	14 (25.0)	
IIIB	4 (9.1)	5 (8.9)	
IV	6 (13.6)	6 (10.7)	

*ANXA9*, annexin A9; UICC, International Union Against Cancer.

**Table III:** Univariate and multivariate analyses for overall survival (Cox’s proportional hazards regression model).

	Univariate analysis			Multivariate analysis		
		
Factors	HR	95% CI	P-value	HR	95% CI	P-value
Age, years (<68/≥68)	5.43	1.42–35.40	0.010*	2.58	0.52–18.92	0.252
Gender (male/female)	1.96	0.58–8.84	0.288			
Histological grade (poor/well-moderate)	0.01	0.00–3.02	0.495			
Tumor size, mm (≥30/<30)	1.13	0.58–2.90	0.731			
Tumor invasion (T4/Tis-3)	2.47	1.39–4.54	0.002*	2.21	1.09–5.02	0.026*
Lymph node metastasis (N1–2/N0)	6.88	2.04–31.12	0.001*	2.07	0.45–10.76	0.342
Lymphatic invasion (present/absent)	5.31	1.39–34.63	0.012*	1.49	0.29–10.93	0.640
Venous invasion (present/absent)	3.78	1.17–12.14	0.026*	2.77	0.77–10.59	0.116
Distant metastasis (M1/M0)	10.82	3.08–35.36	<0.001*	16.64	2.92–132.64	0.001*
*ANXA9* expression (high/low)	3.00	1.32–12.86	0.004*	3.13	1.30–13.69	0.007*

* Statistically significant.

HR, hazard ratio; CI, confidence interval; *ANXA9*, annexin A9.
